# Epidemiology of hepatitis B in Tunisia: retrospective study in 198157 blood donors in military environment

**DOI:** 10.1186/1471-2334-14-S2-P16

**Published:** 2014-05-23

**Authors:** MT Khoufi, A Mrabet, B Nsiri, MN Ebdelli, M Yedeas

**Affiliations:** 1 General Direction of military health, Tunis, Tunisia

## Introduction

Hepatitis B is endemic in Tunisia. His prevalence was evaluated at 5.5% in 1990. The aim of this study is to estimate the prevalence of this disease in the last years in Tunisia.

## Methods

it was a retrospective study about all the blood donors in the Military Center of Blood Transfusion between 2000 and 2011.

The Ag HBs was systematic for every blood donation, by immuno-enzymatic reaction.

## Results

In this period, 198157 blood donors were compiled. These donors were, for 95%, young men, between 20 and 25 years, from all the parts of the country.

This study showed:

• A net decrease in prevalence of hepatitis B in 12 years: from 3.54% in 2000 to 0.8% in 2011.

• A regional disparity of this prevalence.

• No identification of a population at risk.

• The improvement of hygiene and the use of disposable syringes in hospitals can explain this decrease of the prevalence; HVB vaccination began in 1995 and didn’t affect the sample of the study.

## Conclusion

Hepatitis B is still a public health problem in Tunisia. Vaccination and better socio-economic condition will lead in short term to the decrease of the HVB prevalence, started since the 90’s.

